# The advantage of point-of-care ultrasound in central venous catheterization and related pericardial effusion in infants in the NICU

**DOI:** 10.3389/fped.2023.1228070

**Published:** 2023-11-10

**Authors:** Yahui Zhang, Aijing Yan, Yunfeng Liu

**Affiliations:** Pediatric Department, Peking University Third Hospital, Beijing, China

**Keywords:** pericardial effusion, pericardial tamponade, peripherally inserted central catheter, point-of-care ultrasound, umbilical venous catheter

## Abstract

**Background:**

Central venous catheterization (CVC) is broadly used in neonatal intensive care units (*N*ICUs) for efficient vascular access; however, its establishment and maintenance are associated with numerous risks and complications. Here, we focus on investigating the value of point-of-care ultrasound (POCUS) in the early diagnosis and treatment of pericardial effusion associated with CVC and compare the differences in ultrasound and radiography in CVC localization and monitoring in the NICU.

**Methods:**

Twenty-five infants with CVC-associated pericardial effusion (PCE) who were hospitalized in the NICU of Peking University Third Hospital between January 2013 and March 2023 were retrospectively selected for the study. Data concerning their catheterization characteristics, CVC tip position, clinical and imaging manifestations of PCE, treatments, and prognoses were analyzed.

**Results:**

The mean gestational age of our cohort was 29.3 ± 3.1 weeks, and the mean birth weight was 1,211 ± 237 g. The incidence of CVC-associated PCE was 0.65%, and 80% of PCE cases occurred within 4 days of CVC. After PCE, the most common symptoms were tachypnea (44%) and tachycardia (64%). Chest radiographs revealed cardiothoracic enlargement, and only 2 cases (9.10%) showed a “flask heart”. Cardiac ultrasound showed that the catheter tip extended deep into the heart in 72% of infants with PCE. Cardiac insufficiency was observed in 12 cases (48%). Overall, 8 infants (32%) had pericardial tamponade, 7 (87.5%) of whom underwent pericardiocentesis. Overall, 2 (8%) infants died, and the remaining 23 (92%) were cured.

**Conclusion:**

CVC-associated PCE mostly occurs in the early post-catheterization stages (within 4 days) in infants. Some cases may have critical clinical manifestations and progress rapidly, with some even developing pericardial tamponade. A CVC tip being deep into the heart cavity is an important cause of PCE. Compared with chest radiography, point-of-care ultrasound is more accurate for CVC tip positioning and can detect PCE more quickly. Furthermore, it is more advantageous for locating and monitoring CVC-associated PCE. Early identification and diagnosis can effectively reduce fatality rates and improve the prognosis of infants with CVC-associated PCE.

## 1. Introduction

Central venous catheterization (CVC) allows rapid and effective vascular access and is widely used in neonatal intensive care units (NICUs). It is a minimally invasive, safe, and feasible mode of catheterization for infants in the early neonatal period. CVC can effectively reduce the incidence of repeated puncture injuries and peripheral phlebitis, making it an ideal option for mid-to-long-term intravenous infusions in preterm infants. The umbilical venous catheter (UVC) and peripherally inserted central catheter (PICC) are the most widely used (1, 2). Pericardial effusion (PCE) is a CVC-associated complication that can progress to pericardial tamponade. The accumulation of a large volume of fluid in the pericardium can affect the heart's diastolic function. If not identified, diagnosed, and treated in time, this phenomenon can cause severe hemodynamic disturbances with a high mortality risk. Therefore, locating and monitoring CVC are critical and are mainly performed using x-rays and ultrasound examination.

In this study, we retrospectively analyzed the data of 25 infants with CVC-associated PCE who were treated in the NICU of our hospital over the past 10 years. We analyzed their clinical manifestations, catheterization characteristics, treatment strategies, and prognoses. We also compared the differences and accuracies in ultrasound and radiography, to improve the identification and treatment of CVC-associated PCE and choose the best monitoring technique.

## 2. Materials and methods

### 2.1. Study population

This retrospective cohort study included 25 infants who were admitted to the NICU at Peking University Third Hospital after birth and underwent CVC during hospitalization between January 2013 and March 2023. The inclusion criteria were age <28 days and the presence of PCE while a central venous catheter was left in place. The exclusion criteria were PCE detected before catheterization or after catheter removal; the presence of other factors or underlying diseases that led to PCE, such as congenital heart disease, cardiomyopathy, severe anemia, and hypoproteinemia; cardiac insufficiency and rheumatic immune diseases that were present before catheterization; and confirmed intrauterine infections, such as infections caused by *Toxoplasma gondii*, rubella virus, cytomegalovirus, herpes simplex virus, or parvovirus B19. The studies involving human participants were reviewed and approved by the Peking University Third Hospital Medical Science Research Ethics Committee. Written informed consent from the participants’ legal guardian/next of kin was not required to participate in this study in accordance with the national legislation and institutional requirements.

### 2.2. Data collection

Patient data, including basic demographic information; CVC type; time, site, and length of insertion; and catheter position in ultrasound and radiography, were recorded. Data on the time of PCE discovery, clinical manifestation, management measures, ancillary examination, and prognosis were also collected. Cardiography was performed by pediatric physicians with basic training.

### 2.3. The CVC tip position

Numerous authors have suggested that the CVC line tip should be located outside the heart contour. The advocate placement of the PICC tip in the upper limb is the intersection of the superior vena cava and right atrium, with radiographs from the 4th to the 6th thoracic vertebrae. From the lower extremities, the tip of PICC should be located in the inferior vena cava below the thorax, with radiographs from the 9th to the 11th thoracic vertebrae (3). The tip of a UVC should be positioned at the intersection of the inferior vena cava and right atrium (4, 5). With radiographs, the best position is 0.5–1 cm above the diaphragm or at the 9th thoracic vertebra level (6).

### 2.4. PCE diagnosis

The diagnosis was based on echocardiography. PCE diagnosis was confirmed when sonolucent fluid was observed in the pericardial cavity by probing the apical four-chamber, parasternal four-chamber, aortic short-axis, left ventricular long-axis, and subxiphoid four-chamber sections (7). The depths of the sonolucent fluid in the anterior and posterior pericardial cavities, outer region of the wall, and apex of the heart were measured at the end of expiration and ventricular diastole.

### 2.5. PCE grading

PCE grading was based on the results from adults, and the main reference in the neonatal period was PCE distribution. The grading was as follows: mild, when probed in the supine position, with the sonolucent fluid being detected only in the posterior pericardial cavity and not extending to other areas of the pericardial cavity; moderate, when fluid accumulation was observed around the heart, predominantly located in the inferior wall of the left ventricle; and severe, surrounding the entire heart, with a swinging heart sign and clinical signs of cardiac tamponade (8).

### 2.6. Statistical analysis

All analyses were performed using SPSS version 23 (IBM Corp., Armonk, NY, USA). The Kruskal–Wallis test was used for continuous variables. We calculated the mean (±standard deviation) for continuous variables conforming to a normal distribution.

## 3. Results

### 3.1. General information

A total of 3,856 CVC cases in the NICU were retrospectively selected between January 2013 and March 2023, including 1,997 UVC and 1,859 PICC cases. Twenty-five cases of CVC-associated PCE occurred, including 15 and 10 cases associated with UVCs and PICC, respectively. Among those cases, 11 (11/25, 44%) and 14 (14/25, 56%) involved male and female infants, respectively. The mean gestational age was 29.3 ± 3.1 weeks (range, 25–32 weeks), and the mean birth weight was 1,211 ± 237 g (range, 750–1,680) g. In total, 80% (20/25) of PCE cases developed within 4 days (range, 1–4 days) of CVC. The PICC was inserted in the upper and lower extremities in nine and one case, respectively (Table 1).

**Table 1:** General information and condition of the indwelling catheter.

Case	Gestational age (weeks)	Birth weight (g)	Time of insertion (days)	Type of catheter	Site of catheterization	Depth of catheter (cm)	Duration of use
1	32 + 1	1,620	1	PICC	Right basilic vein	10	9 d
2	29 + 6	1,400	5	PICC	Right axillary vein	8	10 h
3	32 + 1	950	4	PICC	Left basilic vein	11	4 d
4	30 + 3	1,120	1	UVC	Umbilical vein	8	3 d
5	29 + 6	1,100	1	UVC	Umbilical vein	7.5	2 d
6	28 + 3	1,100	1	UVC	Umbilical vein	9	2 d
7	29 + 2	1,260	5	PICC	Right basilic vein	12	22 h
8	31 + 1	1,200	2	PICC	Right basilic vein	12	3 d
9	29 + 5	1,040	3	PICC	Right basilic vein	10	4 d
10	32 + 3	1,680	2	PICC	Left basilic vein	10	1 d
11	30 + 4	1,170	1	UVC	Umbilical vein	9	4 d
12	26 + 6	800	1	UVC	Umbilical vein	8	1 d
13	27 + 6	1,050	1	UVC	Umbilical vein	8	1 d
14	29	1,260	1	PICC	Left superficial temporal vein	12	14 d
15	28 + 2	1,350	6	PICC	Left basilic vein	12	1 d
16	29 + 2	1,500	1	UVC	Umbilical vein	9.2	1 d
17	28 + 2	1,320	1	UVC	Umbilical vein	8.5	3 d
18	30 + 5	950	2	PICC	Left great saphenous vein	14	9 d
19	30 + 2	1,350	1	UVC	Umbilical vein	9	1 d
20	25 + 3	750	1	UVC	Umbilical vein	7.5	1 d
21	31 + 2	1,460	1	UVC	Umbilical vein	10	7 d
22	30 + 4	1,170	1	UVC	Umbilical vein	9	4 d
23	31 + 2	1,260	1	UVC	Umbilical vein	8.5	5 d
24	30 + 1	1,160	1	UVC	Umbilical vein	9	1 d
25	28 + 2	990	1	UVC	Umbilical vein	8.5	18 h

d, days; h, hours; PICC, peripherally inserted central catheter; UVC, umbilical venous catheter.

### 3.2. Clinical manifestations

In total, 10 (40%), 14 (56%), 20 (80%), 7 (28%), 4 (16%), and 8 (32%) infants manifested poor reaction, respiratory symptoms, circulatory symptoms, neurological symptoms, digestive symptoms, and other symptoms, respectively. Severe cases may show shock manifestations, such as decreased blood pressure, poor peripheral circulation and perfusion, and multiple organ dysfunction. Five (5/25, 20%) cases had no obvious clinical symptoms or positive signs, and PCE was detected only on routine point-of-care ultrasound (POCUS) examination. Detailed clinical manifestations are presented in Table 2.

**Table 2:** Clinical manifestations in 25 infants with pericardial effusion.

Manifestation	Result	Manifestation	Result
Muffled heart sounds	10 (40)	Poor reaction	10 (40)
Decreased heart rate	7 (28)	Cyanosis	7 (28)
Increased heart rate	16 (64)	Cold hands and feet	10 (40)
Arrhythmia	1 (4)	Prolonged capillary refill time	6 (24)
Decreased blood pressure	6 (24)	Abdominal distention, diminished bowel sounds	4 (16)
Apnea	6 (24)	Gastrointestinal bleeding	1 (4)
Tachypnea	11 (44)	Bruises in both lower extremities	1 (4)
Dyspnea	10 (40)	Localized dermahemia and skin swelling	3 (12)
Pulmonary hemorrhage	1 (4)	Hypotonia	7 (28)
Low urine output	4 (16)	Convulsion	2 (8)

Values represent the number of cases (%).

### 3.3. Chest radiography

A total of 96% (24/25) of infants underwent chest radiography after catheterization. Using vertebral-level assessment, the catheter tips were placed in the correct position in 4 (4/24, 16.67%) cases, at the peripheral depth in 2 (2/24, 8.33%) cases, and too deep in 18 (18/24, 75%) cases. The diaphragmatic level was used to evaluate the position of the UVC tip; in 8 (8/15, 53.33%) cases, the position was correct, and the CVC tips were too deep in 7 cases (7/15, 46.67%). The positions of the CVC tips were adjusted in 11 cases according to x-ray positioning. Chest radiographs were re-examined in 22 (22/25, 88%) infants following PCE development. CVCs were removed in 13 (13/22, 59.10%) infants. Using vertebral-level assessment, the catheter tips were correctly placed in three (3/9, 33.33%) cases but were too deep in six (6/9, 66.67%) cases. Using the diaphragmatic level to evaluate the position of the UVC tip, the CVC tips were correctly placed in three (3/6, 50%) cases, too shallow in one (1/9, 11.11%) case, and too deep in two (2/9, 22.22%) cases. The cardiothoracic ratio increased after PCE development, and the difference was significant (0.53 ± 0.05 vs. 0.60 ± 0.08, *P* < 0.05) (Table 3).

**Table 3:** Ancillary examination of 25 infants with PCE.

Case	Type of CVC	Before PCE						After PCE								
		Chest x-ray			Echocardiogram			Chest x-ray			Echocardiogram					
		CTR	Relative position to the spinal column	Relative position to the diaphragm	Position of the CVC tip	LVEF (%)	Pull-out distance (mm)	CTR	Relative position to the spinal column	Relative position to the diaphragm	Position of the CVC tip	PCE grading	Fluid depth (mm)	LVEF (%)	Compression of the right heart chambers	Swinging heart
1	PICC	0.57	—	—	—	73	—	0.71	—	—	SVC-RA	Massive	3.7	42	Yes	Yes
2	PICC	0.53	T8	—	—	69	—	0.63	T8	—	LA	Massive	5	35	Yes	Yes
3	PICC	0.58	T6	—	SVC-RA	71	—	0.61	—	—	RA	Massive	4	26	Yes	Yes
4	UVC	0.58	T7	10 mm above	RA	76	10	0.65	T7	flat	RA	Massive	6.1	56	Yes	No
5	UVC	0.58	T8	Flat	IVC-RA	67	—	0.61	T8	flat	IVC-RA	Moderate	3	61	No	No
6	UVC	0.57	T7	5 mm above	RA	70	—	0.65	—	—	RA	Moderate	2.8	68	No	No
7	PICC	0.39	T7	—	RA	73	13	0.43	—	—	RA	Moderate	5.1	76	No	No
8	PICC	0.51	T1	—	—	74	—	0.54	—	—	peripheral vein	Moderate	3.4	63	No	No
9	PICC	0.51	T3	—	—	69	—	0.54	—	—	peripheral vein	Massive	10	45	Yes	Yes
10	PICC	0.51	T7	—	LA	71	15	0.46	T5	—	RA	Moderate	10	63	No	No
11	UVC	0.45	T7	Flat	RA	66	—	0.67	T6	8 mm above	RA	Moderate	7	52	No	No
12	UVC	0.5	T7	13 mm above	LA	62	7	0.65	—	—	RA	Massive	11	36	Yes	Yes
13	UVC	0.61	T7	10 mm above	RA	70	—	0.68	—	—	RA	Massive	13	39	Yes	Yes
14	PICC	0.49	T5	—	SVC	60	—	0.48	T4	—	SVC	Moderate	3.6	46	No	No
15	PICC	0.56	T6	—	RA	71	10	0.55	—	—	RA	Moderate	5	68	No	No
16	UVC	0.47	T8	Flat	—	—	—	0.5	T9	4 mm below	IVC	Moderate	3.8	48	No	No
17	UVC	0.59	T8	Flat	IVC-RA	59	—	0.66	—	—	RA	Moderate	4.6	55	No	No
18	PICC	0.53	T9	10 mm below	IVC	64	—	0.57	—	—	IVC	Moderate	3	62	No	No
19	UVC	0.56	T6	16 mm above	—	—	10	0.6	T7	11 mm above	RA	Moderate	4	64	No	No
20	UVC	0.48	T7	10 mm above	RA	57	—	—	—	—	LA	Moderate	6	55	No	No
21	UVC	0.57	T5	15 mm above	LA	61	10	—	—	—	RA	Moderate	8	55	No	No
22	UVC	0.51	T7	11 mm above	—	—	5	0.65	—	—	RA	Moderate	3.7	66	No	No
23	UVC	0.54	T7	14 mm above	—	—	10	0.61	—	—	RA	Small amount	4	62	No	No
24	UVC	0.46	T6	15 mm above	RA	71	10	0.68	T7	12 mm above	RA	Massive	14	53	Yes	No
25	UVC	0.53	T6	13 mm above	RA	61	5	—	—	—	RA	Moderate	9	50	No	No

CTR, cardiothoracic ratio; CVC, central venous catheter; IVC, inferior vena cava; LA, left atrium; LVEF, left ventricular ejection fraction; PCE, pericardial effusion; PICC, peripherally inserted central catheter; RA, right atrium; SVC, superior vena cava; UVC, umbilical venous catheter.

### 3.4. Echocardiogram

In total, 76% (19/25) of infants underwent chest echocardiography after catheterization, which revealed that the catheter tips were correctly positioned in 5 (5/19, 26.32%) cases, deep into the atrium in 12 (12/19, 63.16%) cases, and in the peripheral vein in 2 (2/19, 10.53%) cases. After PCE, all infants underwent ultrasound examination. The positions of the catheter tips were correct in 5 (5/25, 20%) cases, deep into the atrium in 18 (18/25, 72%) cases, and located in the peripheral vein in 2 (2/25, 8%) cases. The left ventricular systolic function was reduced after PCE (67.38 ± 5.51 vs. 53.84 ± 12.09, *P* < 0.05). After PCE, echocardiography revealed a median maximum fluid depth of 4.6 mm (range, 2.8–14.0 mm) and a visible swinging heart sign in 6 (6/25, 24%) cases and indicated left ventricular dysfunction in 11 (11/25, 44%) cases (Table 3).

### 3.5. Clinical treatment

Pericardial tamponade occurred in 8 infants (8/25, 32%, Cases 1–4, 9, 12, 13, and 24). Among them, 7 (7/8, 87.5%) underwent pericardiocentesis. The other 18 (18/25, 72%) received conservative treatments, such as an altered fluid rehydration regimen, CVC removal, fluid volume limiting, and cardiac function enhancement.

### 3.6. Laboratory findings of the pericardial fluid aspirated through pericardiocentesis

In this study, 7 infants (7/25, 28%) underwent pericardiocentesis. The volume of aspirated pericardial fluid was 5–20 ml, and it had a milky-white, red, and light-yellow color in four, two, and one case, respectively. Laboratory tests for the aspirated fluid in Case 4 suggested an increased cell count with mainly lymphocytes and elevated triglyceride levels. In the other 5 cases, laboratory test results suggested a low cell count and lymphocyte percentage, and the glucose concentration was significantly higher than that in the serum (Table 4). All infants were provided constant partial parenteral nutrition infusion by CVC, and antibiotics were administered by CVC to three infants with no history of fluid resuscitation within 12 h.

**Table 4:** Laboratory findings of infants who underwent pericardiocentesis.

Case	Volume (ml)	Characteristic	Specific gravity	WBC (×10^6^/L)	Lym (%)	Glucose (mmol/L)	Protein (g/L)	Chloride (mmol/L)	Triglycerides (mmol/L)	Sudan III staining	Type of infusate	Infusion rate (ml/h)
1	20	Turbid, milky-white	1.012	30	—	23.7	—	—	5.39	Positive	PPN	5.4
2	7	Turbid, milky-white	1.017	2	—	59.8	32	106	10.92	Negative	PPN, amoxicillin clavulanate potassium	3.5
3	5	Turbid, milky-white	—	—	—	—	—	—	—	—	PPN	3.5
4	8	Turbid, milky-white	1.012	116	60	4	<20	108	4.35	Positive	PPN	3.3
12	8	Turbid, red	1.018	353	63	119.3	<4.4	96	—	Positive	TPN, amoxicillin clavulanate potassium, ceftazidime	2.1
13	9	Turbid, yellow	1.015	70	—	83.5	5.2	88	—	Negative	TPN, amoxicillin clavulanate potassium, cefoperazone	2.4
24	5	Turbid, red	1.013	1,252	26	—	—	—	5.93	Positive	PPN	3.1

lym, lymphocytes; PPN, partial parenteral nutrition; TPN, total parenteral nutrition; WBC, white blood cells.

### 3.7. Prognoses and outcomes

Two of the 25 (2/25, 8%) infants died, one of whom had coexisting severe metabolic acidosis, internal environmental disturbances, and acute liver and kidney failure; the infant died 44 h after the disease condition deteriorated. The other one had comorbid respiratory and circulatory failure, severe coagulation disorder, and anemia and died within 3 h of pericardial tamponade development. Among the 23 (23/25, 92%) surviving infants, 6 had pericardial fluid absorption within 4 days after pericardiocentesis. Among the 17 infants treated conservatively, the pericardial fluid was absorbed within 17 days (range, 1–17 days). None of the surviving infants showed structural or functional heart damage during the subsequent follow-up period.

## 4. Discussion

CVC-associated PCE and pericardial tamponade most commonly occur in preterm infants hospitalized in the NICU and are the most common causes of acquired chylous serous cavity effusion (9). In this study, we demonstrate that POCUS is superior to radiographs in terms of CVC tip localization and monitoring, accuracy of identifying complications, and effect of guiding pericardiocentesis. Therefore, monitoring of CVC by POCUS should be popularized in the NICU.

In this study, the incidence rate of CVC-associated PCE was 0.65% (0.75% for UVC and 0.54% for PICC). The mortality rate of infants with pericardial tamponade was 25% (2/8), accounting for 0.02% of CVC cases. Therefore, PCE is a rare complication of CVC, and progression to pericardial tamponade indicates poor prognosis (10). In this study, all neonates were preterm infants with gestational ages of 25–32 weeks and birth weight of 800–1,680 g, suggesting that low birthweight preterm infants are more prone to PCE and/or pericardial tamponade. The pathological mechanism is related to the thin and immature cardiac tissue. In our study, PCE occurred between 10 h and 9 days after birth, and 80% (20/25) of cases developed within 4 days of CVC catheterization, indicating that PCE is more likely to occur in the early post-catheterization stage. Although the CVC tips were too deep based on the radiographic findings in 18 cases, the position of the CVC tip was adjusted in 11 (11/25, 44%) infants after catheterization. However, in 15 (15/25, 60%) infants, the position of the CVC tip had changed during the application. This may have been caused by factors resulting in a large fluctuation in the abdominal circumference in preterm infants, such as physiological weight loss, feeding intolerance, and application of a ventilator. Moreover, limb movement, umbilical cord contracture, and heartbeat can affect the position of the catheter tip (11, 12). Therefore, catheter tip displacement is inevitable, and CVC tip displacement can also lead to PCE development. In this study, echocardiography indicated that the CVC tip was too deep in 18 (18/25, 72%) infants when PCE occurred. CVC-associated PCE can be caused by atrial wall perforation resulting from mechanical injury caused by the CVC tip, but it is most commonly caused by erosion of the right atrial wall (13–15). In this study, the CVC tips were correctly positioned in 5 (5/25, 20%) infants when PCE occurred, with persistent infusion of partial parenteral nutrition, suggesting that persistent infusion of high permeability liquid can damage the endothelial cells and endocardium. The aforementioned can affect vascular permeability and eventually cause PCE (16). Four (4/25, 16%) infants had histories of repeated catheterization, suggesting that repeated penetration of the vena cava by the catheter and multiple irritations of the atrial wall can lead to injury and even micro-perforations, which can ultimately trigger PCE development (15). Therefore, a greater catheter depth is one of the main causes of PCE; parenteral nutrition and repeated catheterization are additional risk factors. Radiographs provide a static, single image to demonstrate the CVC tip position, and it is difficult for infants to maintain an ideal position during the procedure; therefore, detecting alterations of the CVC tip via radiography is not always appropriate. In radiography, inaccurate determinations of the CVC tip may result from difficulties in detecting the landmarks of the superior and inferior vena cava (17). However, infants have great acoustic windows for the examination of the right atrium and a large portion of the superior and inferior vena cava. The relationship of the CVC tip with the heart can be accurately assessed (18). Compared with radiography, echocardiography provides real-time information on CVC tip position, helping to accurately show the catheter tip and the range of position changes associated with limb movement in the ultrasound image (13, 17). Ultrasonic verification of the CVC tip position requires less time and is repeatable and easy to perform (11). This study demonstrates the advantages of echocardiography in accurately detecting the position of PICC tips within the heart. Therefore, we suggest that POCUS can be used for the initial confirmation and follow-up of the CVC tip position.

The increase in the pericardial cavity pressure resulted in a limitation in the cardiac diastolic function, a decrease in cardiac output, and an accumulation of a large fluid volume in the pericardial cavity within a short period, leading to rapid symptom onset. In this study, infants mainly manifested respiratory and circulatory symptoms, rapid progression to multi-organ and multi-system involvement, and internal environmental disturbances (14). Both infants who eventually died had convulsions, which were considered to be related to the development of severe hypoxia, irreversible metabolic acidosis, and internal environmental disturbances. Owing to the lack of typical clinical manifestations in the early stages, PCE identification is often difficult (18). Furthermore, owing to the rapid progression of pericardial tamponade, radiography could not be completed for most infants immediately, resulting in a delayed diagnosis. In this study, radiography suggested that the cardiothoracic ratio of the infants increased after PCE development. However, the majority of the infants had a normal cardiac silhouette and did not show a typical flask-shaped heart. Therefore, radiography is inaccurate and time-consuming, which limits the identification and diagnosis of PCE (19). Conventional radiology cannot be considered the “gold standard” for ECC tip location (20). In contrast, bedside echocardiography can help visualize the catheter position in relation to the heart, allow real-time localization of the catheter tip, identify and evaluate the degree of PCE to confirm the diagnosis, and identify the presence of cardiac compression with pericardial tamponade (21).

In this study, radiographic findings revealed that the catheter was correctly placed in 5 (5/25, 20%) infants, and echocardiography suggested that the tip was deep in the right atrium. The results of the two methods differed, consistent with previous findings (22). Chinese guidelines for central vein catheterization recommend that the central vein catheter tip should preferentially be positioned with radiography, with the landmarks being the thoracic vertebral bodies and the diaphragm. The correct position of the UVC tip is usually 0.5–1.0 cm above the diaphragm or at the 9th thoracic vertebra level (6). These two landmarks are often different, which can be confusing for clinicians. Moreover, the diaphragm is a movable structure that is influenced by factors, such as spontaneous breathing, pulmonary disease, ventilator parameters, and intra-abdominal pressure. These factors contribute to a wide range of motion. Ades et al. reported that catheters properly placed at the right atrial/inferior vena cava junction or in the inferior vena cava, as documented by echocardiography, were located at a wide range of vertebral bodies by the cardiothoracic ratio (T6–T11) (22). The wide variability in atrial size, position, and redundancy of the atrial septum has resulted in no fixed position relationship with the thoracic vertebra. In very low birth weight newborns, correct placement of the UVC tip during radiography does not avoid misplacement by echocardiography (13). Unnecessary removal and attempted replacement of the UVC may result in increased manipulation of the infant, possible loss of central access, and increased radiation exposure. Considering radiation exposure, protection, temperature, and position management, chest radiography in combination with lateral x-ray may be challenging to perform. Therefore, radiography alone is insufficient for determining the adequate position of a CVC, especially in premature infants (13, 22). Ultrasonography enables the quantification of the difference between the tip position, as observed in echocardiography and radiography. POCUS can help visualize the position of the catheter in the heart, allowing the real-time position of the catheter to be identified and evaluated, which has more advantages in CVC positioning (23).

In this study, 8 infants (8/25, 32%) developed pericardial tamponade with severe hemodynamic disturbances, 2 (2/8, 25%) died, and 6 (6/8, 75%) were cured. If an infant with an indwelling CVC develops reduced cardiopulmonary function that cannot be explained by other causes, the possibility of PCE and pericardial tamponade should be considered. Pericardiocentesis can effectively reduce the fatality rate of pericardial tamponade and prevent progression to cardiac arrest (11). In this study, cases of pericardial tamponade were all found by POCUS examination. Seven infants underwent pericardiocentesis under POCUS guidance with an aspirated fluid volume of 5–20 ml. When pericardial tamponade occurred, all infants were provided partial parenteral nutrition infusion at a low speed (2.1–5.4 ml/h) and had no history of liquid expansion in the last 12 h, glucose concentration ≤15%, and osmotic pressure ≤900 mOsm/L. Six infants (6/7, 85.71%) who underwent pericardiocentesis only demonstrated pericardial fluid, and the laboratory test results revealed a low cell count and lymphocyte percentage but a higher glucose concentration than that in the serum, which was inconsistent with the typical characteristics of chyle. Moreover, the effusion did not progress after pericardiocentesis or removal of the central venous catheter. Combined with the pathophysiological findings reported in the literature, these are thought to be associated with the leakage of parenteral nutrient solutions caused by CVC (24). In this study, 17 infants (17/25, 68%) received conservative treatment only, and PCE was absorbed in 1–17 days, suggesting that cases of moderate or low volume PCE with stable hemodynamics can be treated conservatively, with several procedures, such as fluid restriction, applied diuretics, and cardiac stimulants. The surviving infants exhibited no heart structural or functional damage in the long-term follow-up period. Therefore, pericardial effusion can be asymptomatic or a life-threatening event. The abrupt onset of hemodynamic instability without an obvious cause with a CVC *in situ* should raise suspicion of pericardial effusions (20, 25). POCUS can also be used as a localization tool to assist in pericardiocentesis and reduce the exposure of infants to radiation (26). Concurrently, it can reveal changes in ventricular size and cardiac output and identify other heart diseases, such as congestive heart failure. Early identification, diagnosis, and intervention result in positive outcomes and prognoses (27).

Our echocardiography procedure, performed by pediatric physicians with extensive formal training, added strength and high accuracy to our study results. This also revealed that with basic training, implementing POCUS in NICUs is feasible. POCUS can improve the rapid diagnosis of PCE and pericardial tamponage and other critical conditions, reduce radiation exposure, strengthen the continuous monitoring of CVCs (28). Ultrasound can be performed in real time and is the optimal technique for CVC tip positioning in infants (29, 30). Neonatologists can accurately localize the CVC tip and evaluate the hemodynamic and cardiac function (31). However, one of the obstacles to its widespread use is its high operability or dependence. Similar to other technical skills, proper and normalized training and certifications are essential (11). POCUS has not been widely adopted in NICUs in China owing to issues concerning the ultrasound equipment and personnel qualifications, which are also issues in many developing countries. Collaboration with pediatric cardiologists and robust training and accreditation programs are essential to ensure safety and quality service. Therefore, it is essential to provide basic technical training for neonatal physicians, improve cooperation with pediatric cardiology ultrasound specialists, promote the wider application of POCUS in the NICU, and improve its safety in clinical practice.

Our study was limited by its moderate sample size, single-center retrospective nature, and low incidence of CVC-related PCE in the NICU, which may have introduced bias in infant selection and study results. Further research with large samples from multiple centers should be conducted to verify our findings.

In conclusion, CVC-associated PCE mostly occurs in preterm infants with young gestational ages and low birth weights and tends to occur within 4 days after catheterization. It is mostly associated with the CVC tip being located deep inside the atria and has varying clinical manifestations that can rapidly progress to circulatory failure. CVC tip displacement cannot be completely avoided, complications may be fatal, and it is essential to strengthen monitoring. Echocardiography is superior to radiography in the localization and monitoring of CVC. It can obtain anatomical or physiological information to help in making physiology-based decisions or target specific interventions. Regarding CVC and related pericardial effusion, POCUS performed by neonatologists in the NICU offers advantages, such as safety, convenience, rapidity, sensitivity, and high repeatability. POCUS resulted in rapid identification, diagnosis, and timely symptomatic management, significantly reducing diagnosis and treatment duration and improving the clinical prognosis. Thus, POCUS performed by neonatologists in NICUs should become a routine part of clinical practice after standardized training and assessment.

## Data Availability

The original contributions presented in the study are included in the article/Supplementary Material, further inquiries can be directed to the corresponding author.
